# Low-smoke chulha in Indian slums: study protocol for a randomised controlled trial

**DOI:** 10.1186/s12889-017-4369-6

**Published:** 2017-05-16

**Authors:** Megha Thakur, Esther A. Boudewijns, Giridhara R. Babu, Bjorn Winkens, Luc P. de Witte, Jeroen Gruiskens, Preeti Sushama, Cristian T. Ghergu, Onno C. P. van Schayck

**Affiliations:** 1 Department of Family Medicine, Care and Public Health Research Institute (CAPHRI), Maastricht University, P.O. Box 616, 6200 MD Maastricht, the Netherlands; 2 Indian Institute of Public Health Hyderabad-Bangalore Campus, SIHFW premises, 1st cross, Magadi Road, Bangalore, Karnataka -560023 India; 3 Department of Methodology and Statistics, Care and Public Health Research Institute (CAPHRI), Maastricht University, P.O. Box 616, 6200 MD Maastricht, the Netherlands; 4 Centre for Assistive Technology and Connected Healthcare (CATCH), University of Sheffield, 217 Portobello, Sheffield, S1 4DP UK

**Keywords:** Improved cookstove, Air pollution, Respiratory health, Slums, Randomised controlled trial, Protocol

## Abstract

**Background:**

Biomass fuel is used as a primary cooking source by more than half of the world’s population, contributing to a high burden of disease. Although cleaner fuels are available, some households continue using solid fuels because of financial constraints and absence of infrastructure, especially in non-notified slums. The present study documents a randomised controlled study investigating the efficacy of improved cookstove on the personal exposure to air pollution and the respiratory health of women and children in an Indian slum. The improved cookstove was based on co-creation of a low-smoke chulha with local communities in order to support adaption and sustained uptake.

**Methods:**

The study will be conducted in a non-notified slum called Ashrayanagar in Bangalore, India. The study design will be a 1:1 randomised controlled intervention trial, including 250 households. The intervention group will receive an improved cookstove (low-smoke chulha) and the control group will continue using either the traditional cookstove (chulha) or a combination of the traditional stove and the kerosene/diesel stove. Follow-up time is 1 year. Outcomes include change in lung function (FEV_1/_FVC), incidence of pneumonia, change in personal PM_2.5_ and CO exposure, incidence of respiratory symptoms (cough, phlegm, wheeze and shortness of breath), prevalence of other related symptoms (headache and burning eyes), change in behaviour and adoption of the stove. Ethical clearance was obtained from the Institutional Ethics Committee of the Indian Institute of Public Health Hyderabad- Bengaluru Campus.

**Discussion:**

The findings from this study aim to provide insight into the effects of improved cookstoves in urban slums. Results can give evidence for the decrease of indoor air pollution and the improvement of respiratory health for children and women.

**Trial registration:**

The trial was registered with clinicaltrials.gov on 21 June 2016 with the identifier NCT02821650; A Study to Test the Impact of an Improved Chulha on the Respiratory Health of Women and Children in Indian Slums.

**Electronic supplementary material:**

The online version of this article (doi:10.1186/s12889-017-4369-6) contains supplementary material, which is available to authorized users.

## Background

Biomass fuel is used as a primary cooking source by more than half of the world’s population [1, 2]. About three-fourths of this practice occurs in developing countries [1, 3]. Indoor air pollution (IAP) from the use of biomass fuel results in a high burden of morbidity and mortality globally, disproportionately affecting low- and middle-income countries [1, 4]. Exposure is particularly high among women and young children who spend most of their time near the fire [5, 6]. IAP doubles the risk for pneumonia and other respiratory infections for children under the age of 5 years [7]. In addition to respiratory problems, IAP has been linked to tuberculosis, cataracts, preeclampsia/eclampsia, infant mortality, low birth weight and preterm birth [5, 8]. An estimated 70% of Indian households make use of solid fuels [9, 10]. IAP contributes to 4.2% and 6.1% of total morbidity in India, affecting mainly women and children [11]. Although cleaner fuels are available, some groups continue using solid fuels because of financial constraints and absence of infrastructure, especially in non-notified slums [12]. In the short-term, improving cookstoves are often the only affordable solution to reduce IAP [1, 2]. The current EXHALE study initiated by Maastricht University, Zuyd University of Applied Sciences, Indian Institute of Public Health Bangalore, in close collaboration with Narayana Health Hospital and Baptist Hospital located in Bangalore.

## Aims

The aim of the present trial is to reduce the levels of household air pollution and improve the respiratory health of women and children by using a locally designed and manufactured improved cook stove. The study design will be a 1:1 randomised controlled intervention trial.

## Methods

### Study setting

The study area is a non-notified slum called Ashrayanagar in Bangalore, India. This slum is located within 8–12 km from the center of Bangalore. No official record exists on the date of origin of the slum, area covered by the slum, and number of inhabitants. The slum is dynamic in its composition, ranging from concrete houses to basic sheet houses, separated by narrow streets. There are an estimated 1100 households. Most of the households typically cook on traditional three-stone fires (chulha) or kerosene stoves.

### Eligibility criteria

Woman ≥18 years who cooked more than 50% of the meals during the past 30 days (primary cook) and one child per household (0–5 years) will be included in the study. Both households with and without children will be included. When there is more than one child in the age group 0–5 years in a household, one child will be randomly chosen. Women should be capable of being interviewed, and should not migrate in the upcoming 2 months as far as they can predict. Only households cooking solely with a traditional cookstove (chulha) or a combination of a traditional stove and a kerosene/diesel stove will be included. Households with a cooking area outside the house will be excluded. Persons who are seriously ill will be excluded from the study.

### Intervention

The intervention group will receive a locally designed and manufactured improved cookstove (low-smoke chulha). The control group will continue using the traditional cookstove (chulha) or a combination of the traditional stove and the kerosene/diesel stove. The new chulha consists of a primary burner, a secondary burner, a chimney chamber, and a chimney. The improved cookstoves will be installed by field workers. If the stove needs repair during the study, fieldworkers will take care of it.

The first part of project EXHALE was based upon an iterative process of co-creation of a low-smoke chulha with local communities in order to support adaption and sustained uptake. A qualitative study was conducted to gain insight into the cooking practices and challenges faced with the traditional stoves [12]. Workshops were conducted where people were involved in creating an ideal stove, using thermocol blocks. Feedback was continuously used to optimize the design of the cookstove [12]. Improved cookstoves were evaluated in a qualitative study in a slum called Siddhaarthanagar colony in Bangalore. Results of this study will be published elsewhere.

### Outcomes

The primary outcomes include (1) change in lung function (FEV_1_/FVC) over a period of 1 year as measured by spirometry in the primary cook and (2) incidence of pneumonia over the period of 1 year for children ≤5 years. Secondary outcomes include (1) change in personal PM_2.5_ and CO exposure over a period of 1 year for the primary cook (2) incidence of respiratory symptoms, including cough, phlegm, wheeze and shortness of breath over a period of 1 year for the primary cook (3) prevalence of other related symptoms, including headache and burning eyes over a period of 1 year for the primary cook and (4) change in behaviour and attitudes of user and adoption of the improved cookstove over a period of 1 year as part of a process evaluation.

Primary outcomes, including lung function and pneumonia, were selected based on their prevalence in the specific age-groups. Pneumonia is the leading infectious cause of death in children aged 0–5 years and a substantial part might be attributable to IAP [13]. Lung function will be measured to quantitatively assess the relationship between biomass fuel use and lung function impairment.

### Participant timeline

**Fig. 1:**
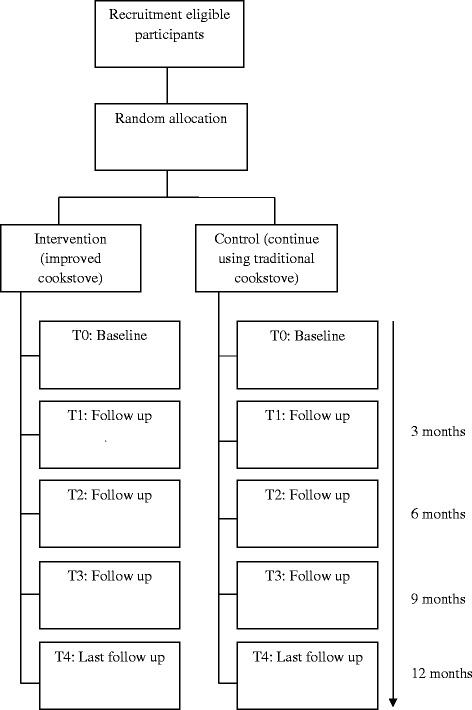
Trial flow-chart

The follow-up time of the study is 1 year (Fig. 1 and Table 1).

**Table 1:** Overview measurements

	FEV_1_/FVC	Pneumonia	Personal PM_2.5_ and CO exposure	Respiratory symptoms	Other related symptoms	Behaviour, attitude and adoption
Baseline (May – July 2017)	X	X	X	X	X	
3 months		X		X	X	
6 months	X	X	X	X	X	X
9 months		X		X	X	
12 months	X	X	X	X	X	X

### Sample size

For adult women, the primary outcome is the mean change in FEV_1_/FVC after 12 months compared to baseline. Based on previous research [14], a standardized effect size of 0.562 is expected, which is a medium to large effect size [15]. To detect this effect size with 80% power and a significance level α of 0.05, 51 adults per group (intervention or control) are required. To account for 25% dropout, the required number of adults is equal to 68 per group. As for children younger than or equal to 5 years, the primary outcome is the proportion of children with pneumonia during the study (within 12 months). Assuming a dropout rate of 25% [16], and a proportion (incidence) of 0.50 within the control group [17], 124 children aged 5 or younger within each group (intervention or control) are required to detect a clinically relevant difference in proportions of 0.20 (proportion of 0.30 in intervention group) with 80% power and a significance level α of 0.05. Therefore, a total of at least 250 households with at least one child aged 5 years or younger will be selected in this study. Next to the child aged 5 years or younger (or the randomly selected child aged 5 or younger if there is more than one eligible child), the adult who is responsible for cooking (mother or someone else) will also be asked to participate. This will ensure that the sample size is also large enough for the adults group (see sample size calculation above). Both eligible households with and without children will be included. The study will be extended to other slums, in case the sample size could not be met in Ashrayanagar slum.

### Recruitment

All houses in the slum area will be mapped manually to create an overview of the study area. Local leaders will be informed about the study and community meetings will be organized. Eligible households will be identified based on inclusion and exclusion criteria. Eligible households will be asked to participate and will be informed about the study procedure. To maximise retention, all households, including the control group, will be informed that they receive the intervention at the end of the study period. Households willing to participate will be asked to sign an informed consent form. In case of children, informed consent will be obtained from mothers or caregivers. Due to high levels of illiteracy common in the slum, an independent witness will be present if eligible participants are illiterate when signing the form.

### Randomisation

Block randomisation will be performed to reduce bias and achieve balance in the allocation of participants to treatment arms [18]. Block sizes vary between two, four and six households to reduce the possibility of knowing the next randomisation allocation. A field team will recruit households. All households participating will receive an ID number, which is given to a non-field member who records the randomisation allocation assigning them to either the intervention or control group. The study will be single-blinded (data-analyst). Randomisation is stratified for having a child aged 5 years or younger or not. A primary cook with a child aged 5 years or younger will be randomised according to the randomisation of the child. Primary cooks without a child aged 5 years or younger will be additionally randomised.

### Data collection

#### Lung function

Change in lung function (FEV_1/_FVC) will be assessed over a period of 1 year as measured by spirometry in the primary cook. Spirometry would be done thrice; at baseline, at 6 months and after 1 year of intervention. Spirometry will be carried out by trained fieldworkers. The highest outcome of three attempts at each point in time will be used in data analysis. A Spirodoc spirometer (MIR, Italy) will be used to assess lung function.

#### Pneumonia

The incidence of pneumonia for children ≤5 years will be determined according to the definition of the WHO Integrated Management of Childhood Illness (IMCI). Pneumonia is defined as cough or difficulty breathing and fast breathing (respiratory rate ≥ 60, ≥50 or ≥40 breaths per minute in those aged <2 months, 2–12 months and 1–5 years respectively). Severe pneumonia is defined as cough or difficulty breathing and chest-indrawing, stridor or any general danger signs. General danger signs include inability to drink or breastfeed, vomiting, convulsions, lethargy, or unconsciousness [19].

Screening for pneumonia will be done every 3 months for 1 year. Physical examination will be conducted by trained fieldworkers.

#### Air pollution

Personal air pollution devices were developed by a local company called Cingularity TEC India Pvt. Ltd. This device measures both PM_2.5_ and CO levels. Personal exposure to PM_2.5_ and CO will be measured for the primary cook two times a week for 24-h hours (total 48-h hours), at baseline, after 6 months and after 12 months.

Ambient air pollution will be measured to control the environmental effects. Two stationary outdoor samples will be placed in the slum for monitoring ambient air pollution. Data will be collected on a continuous basis for 1 year.

#### Questionnaires

Three types of questionnaires will be used. The first questionnaire contains items to map demographic data and to scan for confounders and effect modifiers, including active smoking behaviour of fathers in houses. Pregnancy and type of stove used will be recorded. The data will be used for evaluating exposure to IAP. The information will be collected for all participants at baseline. The second questionnaire assesses symptoms of respiratory morbidity, including cough, phlegm, wheeze, shortness of breath, and symptoms of exposure to air pollutants such as burning eyes and headaches for the primary cook. All other symptoms or conditions considered to be relevant by participants will be recorded. The information will be collected once every 3 months for 1 year. The third questionnaire will include questions regarding changes in behavior, attitude of users and compliance of the primary cook. The questionnaire will be conducted twice a year; at 6 months and after 1 year. All questions will be asked in the local language (Kannada). Questionnaires are displayed in Additional file 1.

#### Interviews

The qualitative semi-structured interview will assess the change in behaviours, attitudes among women and the perceived barriers and facilitators to adoption of the improved cookstove. Interviews will be conducted twice; after 6 months and after 1 year of intervention in a sub-group of the participants who received the intervention. Information will be collected until saturation is reached. All questions will be asked in the local language (Kannada). The interview guide is displayed in Additional file 2.

#### Indicator smoke and soot

In households participating in the qualitative interviews, a standardized piece of white plasterboard will be used to indicate smoke and soot escaped from the stove. The plasterboard of a slightly larger size than the surface covered by the chulha will be placed above the hobs on the wall at the same site to which the stove is installed. The colour of the plasterboard will be used as a qualitative outcome and as educational material for the primary cook.

### Data management

Data for the questionnaires and for the assessment of pneumonia will be collected using a tablet computer with a pre-formatted questionnaire sheet. Data will directly be transferred to an online cloud. The system uses 128-bit SSL secured layer for all data transactions. Both the software subsystem and the database system are secured by this layer. The system can only be accessed through secured username and password. The principle of least privilege, whereby access will be limited to the minimal level that will allow normal functioning, will be followed. Interviews will be audio-recorded and a transcript will be made by fieldworkers after the visits. Data will be stored on a hard-drive. Personal air pollution measurements will be stored on an SD-card in the device and ambient air pollution measurements will be stored on an SD-card and in an online website. Results of spirometry will be transferred to the software winspiroPRO after every visit and will be stored on a hard-drive. Back-ups will be made for all measurements to avoid loss of data. All data collection and storage devices will be password protected. Identifiers, data, and keys will be placed in separate, password protected/encrypted files. Only supervisors and the main researcher will have access to the data files, identifiers, and keys. All data will be stripped of identifiers as soon as possible, and managed by use of a code.

### Data analysis

An intention-to-treat analysis will be conducted in order to ensure external validity of the study and minimize bias. For missing observations, multiple imputation method will be used. Mean (standard deviation) or median (interquartile range IQR, i.e. 25th percentile – 75th percentile) where appropriate will be used for numerical variables, while categorical variables will be presented by number of participants or households (percentage, %). Linear and logistic mixed model analyses will be used to assess the impact of the intervention on numerical and categorical outcomes that are measured repeatedly, respectively. These methods will be used, since they take the correlation between repeated measures into account. For numerical and categorical outcomes that are only measured once, linear and logistic regression analysis will be used. Analyses will include the stratification variable next to grouping variable indicating the treatment allocation (intervention or control) and will be carried out with and without covariates that are related to the outcome, including personal, household and community characteristics. To address potential effect modifiers (X), variables will be added to the model as interaction terms. One potential effect-modifier will be considered, namely active smoking behaviour of fathers in houses.

Interviews will be audio-recorded and be transcribed in Kannada (local language). Transcripts will be translated into English. An inductive coding procedure will be applied, whereby themes derive from emergent patterns in the data. Each transcript will be independently coded by two researchers. Final coding will be based on consensus, with arbitration by a third researcher in the case of disagreement. All transcripts will be manually coded. Ultimately the data will be analysed according to the themes and relevant quotes and text will be consolidated and summarized.

### Data monitoring

Since the intervention is not a clinical intervention, and does not have adverse effect on the participants, a data monitoring committee has not been formed. However, to review and keep track of the progress of the trial, a clinical advisory committee comprising of relevant experts has been formed. The research team and the members of the clinical advisory committee will meet periodically to review the trial progress. Since the trial does not involve any drugs or medical procedures, the advisory committee is not carrying out any interim analysis.

## Discussion

As far as we know, no studies on improved cookstoves have been conducted in deprived urban areas, especially not in slums, while the effects might be considerably different between rural and urban settings [11]. The findings of this study will assess the effects of an improved cookstove intervention in deprived urban areas. The results may provide evidence for the decrease of air pollution due to improved cookstoves and give critical information about the health effects for both children and women. There can be significant public health implications of the study. First of all, results can directly inform the potential impact of the dissemination of improved cookstoves in (Indian) slums. Secondly, results on the feedback of the improved cookstove can be used to improve the development of the stove. Lastly, the study can be used as a basis for scaling up interventions in other low- and middle income countries in the world.

### Recruitment status participants

Ongoing.

## Additional files

Additional file 1: Questionnaires. (PDF 350 kb)

Additional file 2: Interview guide. (PDF 152 kb)

## Abbreviations

CO: Carbon monoxide
FEV_1_: Forced expiratory volume in first second
FVC: Forced vital capacity
IAP: Indoor air pollution
PM: Particular matter
WHO: World Health Organization
