# Supplementary material for: Low-smoke chulha in Indian slums: study protocol for a randomised controlled trial
Source: BMC Public Health. 2017 May 16;17:454. doi: 10.1186/s12889-017-4369-6 (PMC5434517; doi:10.1186/s12889-017-4369-6)
Supplement: Supplementary file 1 — Questionnaires. (PDF 350 kb) [file 12889_2017_4369_MOESM1_ESM.pdf]

Questionnaire 1: Baseline household demographic characteristics
For primary cook

Remarks:<br>1) If there is a multiple choice question, all choice options must be read to the participant to allow him to select an option<br>2) The participant can always choose for the option 'no response' or 'not known'<br><br>The participant has signed the informed consent form and opportunity has been given to ask questions and clear up any doubts<br>*(confirm in order to continue)*

General information
Household ID (*to be filled by interviewer):*
Person ID (*to be filled by interviewer):*
Name:
Name interviewer (*to be filled by interviewer):*
Date of interview (*to be filled by interviewer):*

Season of the year (*observation interviewer):*
-Rainy (it has rained in the last two weeks)
-Dry (it has not rained in the last two weeks)

Date of birth of eligible women: ___ / Not known/ No response
If not known → age of eligible woman: ___ years / Not known/ No response

Current marital status: married, irrespective of whether the spouse lives in the same household/ married, but husband is dead/ married, but divorced / never married

Are you pregnant? Yes / No/ Not known (not sure) / No response
Yes→How many months pregnant are you, or if you are not sure, how many months do you believe you might be? _____ months / not known / no response

Family size
- number of men (18 years and older): __ /No response
- number of women including yourself (18 years and older): __ /No response
- number of children (17 years and younger): ____ /No response

Socio-economic status
Religion of the household: Hindu/ Muslim/ Christian/ Sikh/ Jain/ Parsi/ No religion/ Others (specify)/ No response

Caste of the household: General/ Sch. Caste/ Sch. Tribe/ Other backward class/ Others (specify)/ No response

Education of eligible woman: Illiterate/ not completed primary school/ primary school completed/ middle school completed / high school completed / intermediate or post high school completed / professors or honours

Current occupation:

-construction worker
-garbage collector
-housekeeper (office/company/mall etc.)
-domestic worker
-garments industry
-self-employed (specify) ______
-unemployed/no occupation
-other (please specify) ______
-No response

What is the average monthly income of your household?
-<1865 INR
-1866-5546 INR
-5547-9248 INR
-9,249-13,873 INR
-13,874-18,497 INR
-18,498-36,996 INR
->=36,997 INR
-Not known
-No response

What type of ration card does the household have? BPL card/ APL card/ Anthyodaya card/ Not known/ No response

The house where you live is: not rented, but also not owned / borrowed / rented / owned / other (specify) / not known / no response

Does your household have:

	Yes=1	No=2		Yes=1	No=2
Electricity			Internet		
Mattress			Computer		
Pressure cooker			Refrigerator		
Chair			Air conditioner/cooler		
Cot/Bed			Washing machine		
Table			Watch/clock		
Electric fan			Bicycle		
Radio/transistor			Motorcycle/scooter		
B&W television			Animal-drawn cart		
Colour television			Car		
Sewing machine			Water pump		
Mobile telephone			Thresher		
Land line telephone			Tractor		

*B&W television = Black and white television

Do you own a toilet? Yes/ No/ No response

What kind of toilet facility do members of your household usually use? Flush toilet/ pour flush toilet (water poured by hand)/ Pit latrine/ No facility; uses open space or public toilet/ Not known/ No response

Which health facility does your family refer to when needed? Government hospital/ ESI/ private hospital/ private clinic/ local healer/ others (specify)/ not known/ no response

House characteristics

How many rooms do you have in your house (*observation interviewer)*?
- A single structure where the cooking area is the same area (room) as the rest of the living areas/bedroom

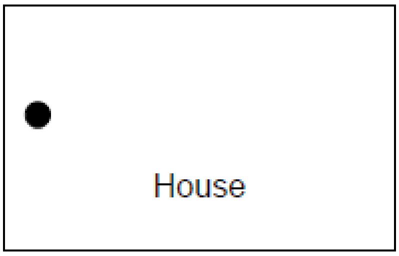

- A single structure with the area for cooking located in another room and separated **by a partial wall** from the other main areas and/or bedroom

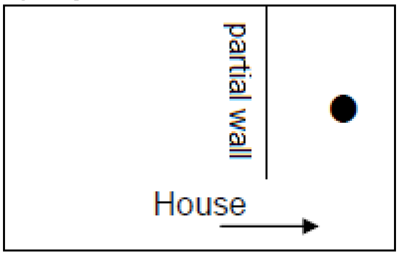

- A single structure with the area for cooking located in another room and separated **by a complete wall** (covered till the roof) from the other main areas and/or bedroom

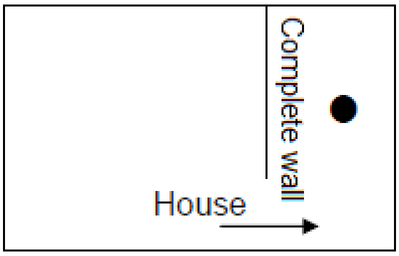

Where do you sleep? Same room as the kitchen/ In a room partially separated from the kitchen/ In a room completely separated from the kitchen / no response

Main material of the roof (*observation interviewer):* Thatch (palm leaf, grass etc)/ Sheet/ Tile/ Concrete/ Others (specify)

Main material of the floor (*observation interviewer):* Mud/ dung/ brick/ tile/ cement/ others (specify)

Main material of the wall (*observation interviewer):* Sheet/ Mud/ Brick/ Concrete/ Others (specify)

The spaces between walls and roof are (*observation interviewer)*: completely closed/ partially open

Leaks in roof (*observation interviewer):* Yes/ No

Number of windows (*observation interviewer):*
Size of window (*observation interviewer):*

Window 1	cm
Window 2	cm
Window 3	cm

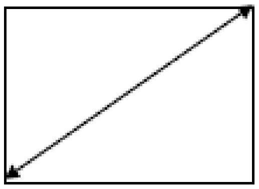

**(Measure the window diagonally as shown in the drawing above and write down measurements in centimeters)

Numbers of doors (*observation interviewer):*

Stove and fuel

I am going to ask you some questions about cooking and about your stove. You might have another stove for boiling water that you use for example bathing. The questions are only about the stove that you use for cooking food or making coffee or tea.

Type of cookstove you are using: traditional/ kerosene or diesel/ combination of traditional and kerosene or diesel stove/ other (specify)

How long have you been using the current stove? __ years / not known / no response

Traditional stove	years
Kerosene or diesel stove	years

Shortest distance of cookstove from the doors or windows (*observation interviewer)*: __ meter

Height of the stove (*observation interviewer*): _____ cm

How many times a day do you light the stove for cooking? ___/ not known / no response

How much time do you spend cooking in total per day? (Hours/day) __ / not known/ no response

How much time do you spend inside the house on average?
-Almost the entire day (more than 80% of the time)
-During the nights and evenings and sometimes during the day
-During the nights and evenings
-Only during the nights

-Other (specify)
-Not known
-No response

Do you keep the windows or doors open when you cook? Yes, always / Yes, most of the times (more than 80% of the time)/ Sometimes (<80% of the time) / No/ Not known/ No response

Type of fuel used for cooking

Type of fuel used	What fuel are you using the most? And the 2nd most? (etc.) 1 = most used; 2 = second most used (etc.) / no response	Do you buy (=1) or collect (=2) or buy and collect (=3) this fuel?	Buy → How much do you pay for this fuel per week? / not known/ no response	Collect → How much time do you spend each week collecting this fuel (hours)? / not known/ no response
Wood			INR	
Kerosene			INR	
Diesel			INR	
Rubbish (paper, plastic etc.)			INR	
Coconut-shell			INR	
Charcoal			INR	
Other (specify)			INR	

Wood → How often are you using dry/damp wood **for cooking** on a scale from 1 to 5

1= my wood is always damp
2= most of the time, my wood is damp
3= half of the time, my wood is damp and half of the time, my wood is dry
4=most of the time, my wood is dry
5=my wood is always dry

Do you have a chimney? (*observation interviewer)*: Yes / No

Do you sometimes **cook** outside your house? Yes/ No/ No response
Yes → During what season of the year? Dry (it has not rained in the last two weeks) / Rainy (it has rained in the last two weeks)
Yes → How many times a day do you light the stove for cooking outside? 1 / 2 / 3 / ≥4 / not known/ no response

I am going to ask you a question about the stove you are using for boiling water, for example for bathing.

Are you using another stove for **boiling water**? Yes / No / No response
Yes → Do you boil water outside or inside the house? Inside / Outside/ No response
Yes → What kind of fuel are you using for boiling water? Wood/ Kerosene/ Diesel/ Rubbish (paper, plastic)/Coconut-shell/charcoal/other (specify)/ not known/no response

Other sources of exposure
How often do you burn a kerosene lamp in your house? Never/ less than once per week/ one to two times per week/ three to four times per week/ five or more times per week/ not known/ no response

Do you smoke or have you smoked cigarettes/bidis? Never smoked / used to smoke, not anymore / smoke now / no response

Used to smoke → How long ago did you quit smoking (years)? __ / not known / no response

Smoke now → Approximately how many cigarettes/bidis do you smoke per day? __ / not known/no response

Smoke now → Do you smoke **inside the house**? Never / occasionally / almost every day but only a few cigarettes/bidis (up to 2) / almost daily many cigarettes/bidis (more than 2) / no response

We are now going to ask you some questions about the smoking behaviour of men in your household.

	Does he smoke or has he smoked cigarettes/bidis? Never smoked / used to smoke, not anymore / smoke now / not known/ no response	Used to smoke→ How long ago has he quit smoking? (years)/ not known/ no response	Smoke now → Does he smoke **inside the house**? Never / occasionally / almost every day but only a few cigarettes/bidis (up to 2) / almost daily many cigarettes/bidis (more than 2) / not known/ no response
Man 1			
Man 2			
Man 3			
Man 4			

Medical status
Have you ever been diagnosed with :

Disease	Yes / no / not known/ no response	Yes → are you cured now? Yes/No/not known/no response	Yes → Do you receive any treatment or medications? Yes/No/not known/no response
Asthma			
COPD			
Allergies			
Tuberculosis			
Heart condition			

Did you visit any health centers during the past 1 year? Yes/ No / not known/ no response
Yes → For what reason? __ / not known / no response
Yes → Were you also admitted? Yes/ No / not known/ no response
Yes → For how long did you stay in the hospital? __ days / not known / no response

Is there any information that you would like to share with us? ___ / not known/ no response

Thank participant for cooperation – End interview

Questionnaire 1: Baseline household demographic characteristics
For children (0-5 years)

Remarks:<br>1) **The mother will be asked to complete the questionnaire (please fill in the name and person ID of the child in general information; not name of the mother)**<br>2) If there is a multiple choice question, all choice options must be read to the participant to allow him to select an option<br>3) The participant can always choose for the option 'no response' or 'not known'<br><br>The participant has signed the informed consent form and opportunity has been given to ask questions and clear up any doubts<br>*(confirm in order to continue)*

General information
Household ID (*to be filled by interviewer):*
Person ID of child (*to be filled by interviewer):*
Name of child:
Name interviewer (*to be filled by interviewer):*
Date of interview (*to be filled by interviewer):*

Date of birth: ___ / Not known/ No response
If not known → age: ___ years / Not known/ No response

Sex: boy / girl / no response

Other sources of exposure
While you are cooking, where is your child most of the time?
-On my knees
-Inside the house, but not on my knees
-Outside
-In another place (specify)
-Not known
-No response

Where does your child sleep? Same as kitchen/ Separate room but same house as kitchen / no response

Medical status
Has your child ever been diagnosed with :

Disease	Yes / no / not known/ no response	Yes → are you cured now? Yes/No/not known/no response	Yes → Do you receive any treatment or medications? Yes/No/not known/no response
Asthma			
Allergies			
Tuberculosis			
Heart condition			

Did your child visit any health centers during the past 1 year? Yes/ No / not known/ no response
Yes → For what reason? __ / not known / no response
Yes → Was your child also admitted? Yes/ No / not known/ no response
Yes → For how long did your child stay in the hospital? __ days / not known / no response

Is there any information that you would like to share with us?

Thank participant for cooperation – End interview

Questionnaire 2: respiratory health
For primary cook

Remarks:<br>1) If there is a multiple choice question, all choice options must be read to the participant to allow him to select an option<br>2) The participant can always choose for the option 'no response' or 'not known'<br><br>The participant has signed the informed consent form and opportunity has been given to ask questions and clear up any doubts<br>*(confirm in order to continue)*

General information
Household ID (*to be filled by interviewer):*
Person ID (*to be filled by interviewer):*
Name:
Name interviewer (*to be filled by interviewer):*
Date of interview (*to be filled by interviewer):*

- Cough
Have you had a cough in the past month? Yes/No/ Not known/ No response
Yes → Do you usually cough 4 or more times a day for several days in a week? Yes/No/not known/no response
Yes → Do you usually cough when you wake up in the morning? Yes/No/not known/no response
Yes → Do you usually cough during the rest of the day or at nights? Yes/No/not known/no response
Yes → Did you cough prolong for 3 continuous months or more during the past 1 year? Yes/No/not known/no response

- Phlegm
Have you had phlegm from your chest (not from your nose) in the past month? Yes/No/ Not known/ No response
Yes → Do you usually bring up phlegm 2 or more times a day for several days in a week? Yes/No/not known/no response
Yes → Do you usually bring up phlegm when you wake up in the morning? Yes/No/not known/no response
Yes → Do you usually bring up phlegm during the rest of the day or at nights? Yes/No/not known/no response
Yes → Did you bring up phlegm for 3 continuous months or more during the past 1 year? Yes/No/not known/no response

- Shortness of breath:
Are you troubled by shortness of breath when hurrying or walking a staircase? Yes/No/ Not known/ No response
Do you have to walk slower than people of your age because of breathlessness? Yes/No/ Not known/ No response
Do you ever have to stop for breath when walking at your own pace? Yes/No/ Not known/ No response

Do you sometimes have to stay at home because you can't go out of the house because of breathlessness?

-Wheezing
Have you had wheezing or whistling in the chest in the past month? Yes/No/ Not known/ No response
Yes → in the last month, how often, on average has your sleep been disturbed due to wheezing? Never woken with wheezing/ Less than one night per week/ one or more nights per week
Yes → In the last month, have you had wheezing or whistling in the chest even without having a cold or flu?
Yes → In the last month, how much did wheezing interfere with your daily activities on a scale from 1 to 5?
1=I can't do anything due to wheezing
2=Most of the times, I can't do anything due to wheezing
3=Sometimes, I can't do anything due to wheezing
4=Most of the times, it doesn't interfere with my daily activities
5=It doesn't interfere my with my daily activities at all

- Headache
During the past month, have you had headaches? Yes/ No/ Not known/ No response
Yes → How often do you have headaches? __ times/week
Yes → How painful are the headaches on a scale from 1 to 5?
1=Very severe pain (disabling; I can't perform daily activities)
2=Severe pain (interferes with daily activities)
3=Moderate pain (interferes a little bit with daily activities)
4=Mild pain (nagging, annoying)
5=No pain

-Burning eyes
During the past month, have you had burning eyes and/or water eyes? Yes/ No/ Not known/ No response
Yes → How often do you have burning eyes and/or water eyes? __ times/week
Yes → Do you have a yellow or greenish and sticky substance coming out of your eyes? Yes/ No/ Not known/ No response

- Smoke from neighbors
Does the smoke from your neighbors enter your house? Yes, almost always (80% of the time or more) / Yes, sometimes / No/ Not known / No response
Yes → How far is their smoke producing place from your house? (*observation interviewer)*: __ meters

In the last week, did you visit your neighbor's house while they were cooking? Yes/No/Not known/No response
Yes → what kind of stove do they have? Traditional/ New cook stove/ Kerosene or diesel stove/ LPG (multiple answers possible)
Yes → How often have you visited them while they were cooking? __ times/week

Do not ask the following question during baseline:
Are you pregnant? Yes / No/ Not known (not sure) / No response

Yes→How many months pregnant are you, or if you are not sure, how many months do you believe you might be? _____ months / not known / no response

Do not ask the following question during baseline:
Do you smoke or have you smoked cigarettes/bidis? Never smoked / used to smoke, not anymore / smoke now / no response

Used to smoke → How long ago did you quit smoking (years)? __ / not known / no response

Smoke now → Approximately how many cigarettes/bidis do you smoke per day? __ / not known/no response

Smoke now → Do you smoke **inside the house**? Never / occasionally / almost every day but only a few cigarettes/bidis (up to 2) / almost daily many cigarettes/bidis (more than 2) / no response

Do not ask the following question during baseline:

We are now going to ask you some questions about the smoking behaviour of men in your household.

	Does he smoke or has he smoked cigarettes/bidis? Never smoked / used to smoke, not anymore / smoke now / not known/ no response	Used to smoke→ How long ago has he quit smoking? (years)/ not known/ no response	Smoke now → Does he smoke **inside the house**? Never / occasionally / almost every day but only a few cigarettes/bidis (up to 2) / almost daily many cigarettes/bidis (more than 2) / not known/ no response
Man 1			
Man 2			
Man 3			
Man 4			

Do you have any other complaints or symptoms you would like to share with us?

Thank participant for cooperation – End interview

Questionnaire 3: changes in behavior and attitude of users
For primary cook

Remarks:<br><br>1) If there is a multiple choice question, all choice options must be read to the participant to allow him to select an option<br>2) The participant can always choose for the option 'no response' or 'not known'<br><br>The participant has signed the informed consent form and opportunity has been given to ask questions and clear up any doubts<br>*(confirm in order to continue)*

General information
Household ID (*to be filled by interviewer):*
Person ID (*to be filled by interviewer):*
Name:
Name interviewer (*to be filled by interviewer):*
Date of interview (*to be filled by interviewer):*

Season of the year (*observation interviewer):*
-Rainy (it has rained in the last two weeks)
-Dry (it has not rained in the last two weeks)

<u>General information on cooking</u>
How many times a day do you light the stove for cooking (not boiling water) ___/ not known / no response

How much time do you spend cooking in total? (Hours/day) __ / not known/ no response

Did you keep the windows and doors open when you cook? Yes, always / Yes, most of the times (more than 80% of the time)/ Sometimes (<80% of the time) / No/ Not known/ No response

How much time do you spend inside the house on average?
-Almost the entire day (more than 80% of the time)
-During the nights and evenings and sometimes during the day
-During the nights and evenings
-Only during the nights
-Other (specify)
-Not known
-No response

Only for intervention group

I am going to ask you some questions about cooking and about your stove. You might have another stove for boiling water that you use for example bathing. The questions are only about the stove that you use for cooking food or making coffee or tea.

Type of cookstove

What type of cookstove are you using for cooking? (multiple answers possible)

Type	Tick all that apply	Yes → Some of the cooking (one meal)/ half of the cooking/ most of the cooking (2-3 meals)/ all of the cooking/ not known/no response	Yes → Outside (=1) or inside (=2) the house?
New stove			
Traditional			
Kerosene/diesel			
LPG			
Other (specify)			

Type of fuel used for cooking

Type of fuel used	What fuel are you using the most? And the 2nd most? (etc.) 1 = most used; 2 = second most used (etc.) / no response	Do you buy (=1) or collect (=2) or buy and collect (=3) this fuel?	Buy → How much do you pay for this fuel per week? / not known/ no response	Collect → How much time do you spend each week collecting this fuel (hours)? / not known/ no response
Wood			INR	
Kerosene			INR	
Diesel			INR	
Rubbish (paper, plastic etc.)			INR	
Coconut-shell			INR	
Charcoal			INR	
LPG			INR	
Other (specify)			INR	

Wood → How often are you using dry/damp wood for cooking on a scale from 1 to 5?
1= my wood is always damp
2= most of the time, my wood is damp
3= half of the time, my wood is damp and half of the time, my wood is dry
4=most of the time, my wood is dry
5=my wood is always dry

<u>Only for use of new stove, but not exclusively (intervention group)</u>

<u>Barriers</u>

Why do you not use the new cookstove exclusively? *Now I'm going to mention some possible reasons for not using the new cookstove exclusively. Please indicate if that applies to you. You can have multiple reasons.*

Reason	Tick all that apply
It takes too long to cook my food	
My pots do not fit on the stove	
The stove is broken or does not work well	
More smoke than the traditional stove	
I don't know how to use it/I don't feel comfortable using it	
Food doesn't taste the same	
I am afraid my child will get burned	
It consumes more wood	
Other (specify)	
Not known	
No response	

Only for non-users (intervention group)

Barriers

When did you switch to another stove? Less than 2 months ago/ more than 2 months ago / not known / no response

Less than 2 months ago → Why did you stop using the new cookstove? *Now I'm going to mention some possible reasons for not using the new cookstove exclusively. Please indicate if that applies to you. You can have multiple reasons.*

Reason	Tick all that apply
It takes too long to cook my food	
My pots do not fit on the stove	
The stove is broken or does not work well	
More smoke than the traditional stove	
I don't know how to use it/I don't feel comfortable using it	
Food doesn't taste the same	
I am afraid my child will get burned	
It consumes more wood	
Other (specify)	
Not known	
No response	

Less than 2 months ago → Did you remove the stove from your house? Yes / No / No response

Less than 2 months ago → Go to attitude regarding new stove

Only for users of the new stove (exclusively and non-exclusively) (intervention group)

Behavior and environmental changes

Did you remove the chimney from the new stove? Yes/ No/ No response

Did you raise the height of the stove? Yes/ No/ No response

Did you change the position of the new stove? Yes/ No/ No response

Did you seal the new stove with mud? Yes/ No/ No response
Yes → How often? Only once / 1-2 times a month/ 3-4 times a month/ more than 4 times a month / Not known/ No response

Did you clean the chimney of the new stove? Yes/ No/ No response
Yes → How often? Only once/ 1-2 times a month/3-4 times a month/more than 4 times a month / Not known/ No response

Did you cover the second hob when you were cooking on the primary hob?
Yes, always / Yes, most of the times (more than 80% of the time)/ Sometimes (<80% of the time) / No/ Not known/ No response

<u>Only for use of new stove (exclusively and non-exclusively) and no use of new cookstove within 2 months of switch (intervention group)</u>

<u>Stove damage and repair</u>
Did your new stove break during the last two months? Yes/No/No response
Yes → What part of your new stove was damaged? Chimney/cracks in first chamber/ cracks in second chamber/ other (specify) / No response
Yes → Did you not use your new stove because it broke? Yes/ No / No response
Yes → For how long did you not use your new stove? ____ weeks
Yes → Was your stove repaired? Yes/ No / No response

<u>Attitudes regarding new stove</u>
How happy are you with the new stove on a scale from 1 to 5?
1=I am totally not happy with the stove
2=I am not happy with the stove
3=neutral; I am not happy, but also not unhappy
4=I am happy with the stove
5=I am very happy with the stove
No response

What do you think about the durability of the new stove? I think it will only last for one month/ I think it will last for six months/ I think it will last for 1 year/ It think it will last for more than 1 year/ Not known/ No response

What do you think about the appearance of the new stove on a scale from 1 to 5?
1=It is very ugly
2=It doesn't look nice
3=Neutral; it is not ugly nor beautiful
4=I looks nice
5=It is beautiful
No response

How do you feel about the fuel consumption of the new stove on a scale from 1 to 5?
1=It consumes a lot more fuel than my previous stove
2=It consumes a little bit more fuel than my previous stove
3=It consumes the same amount of fuel as my previous stove
4=I consumes a little bit less fuel than my previous stove
5=It consumes a much less fuel than my previous stove
No response

How do you feel about the smoke production of the new stove on a scale from 1 to 5?
1=It produces much more smoke than my previous stove
2=It produces a little bit more smoke than my previous stove
3=It produces the same amount of smoke as my previous stove
4=I produces a little bit less smoke than my previous stove
5=It produces much less smoke than my previous stove
No response

How comfortable are you using the new stove on a scale from 1 to 5?

1=It is very difficult to use the stove
2=It is difficult to use the stove
3=It is not easy, but it is also not difficult
4=It is easy to use the stove
5=It is very easy to use the stove
No response

How much time (more or less) do you spend on cooking using the new stove in comparison with the previous stove on a scale from 1 to 5?
1=It takes a lot more time to cook my food
2=It takes a little bit more time to cook my food
3=It takes the same time to cook my food
4=It takes a little bit less time to cook my food
5=It takes much less time to cook my food
No response

Does the stove allow you to use different sizes of pots? Yes/ No / Not known/ No response

How is the taste of the food cooked with this stove? It is the same as cooked on the previous stove / It is better than the food cooked on the previous stove / it is worse than the food cooked on the previous stove/ No response

How would you rate the safety of the new stove on a scale from 1 to 5?
1=It is very dangerous
2=It is a little bit dangerous
3=It is not dangerous, but also not safe
4=It is safe most of the times
5=It is completely safe
No response

Do the following characteristics influence your decision to use the stove on a scale from 1 to 5?
1=It keeps me away from using the stove
2=I don’t like it much, but it doesn’t keep me away from using the stove
3=It doesn’t influence my decision
4=It do like it, but it doesn’t really motivate me to use the stove
5=It motivates me to use the stove

	1	2	3	4	5
Durability					
Appearance					
Fuel consumption					
Smoke production					
Comfort					
Time to cook food					
Use of different pots					
Taste of food					
Safety					

Thank participant for cooperation – End interview

<u>Only for control group</u>

I am going to ask you some questions about cooking and about your stove. You might have another stove for boiling water that you use for example bathing. The questions are only about the stove that you use for cooking food or making coffee or tea.

Type of cookstove you are using for cooking
(multiple answers possible)

Type	Tick all that apply	Yes → Some of the cooking (one meal)/ half of the cooking/ most of the cooking (2-3 meals)/ all of the cooking/ not known/no response	Yes → Outside (=1) or inside (=2) the house?
Traditional			
Kerosene/diesel			
LPG			
Other (specify)			

Type of fuel used for cooking

Type of fuel used	What fuel are you using the most? And the 2nd most? (etc.) 1 = most used; 2 = second most used (etc.) / no response	Do you buy (=1) or collect (=2) or buy and collect (=3) this fuel?	Buy → How much do you pay for this fuel per week? / not known/ no response	Collect → How much time do you spend each week collecting this fuel (hours)? / not known/ no response
Wood			INR	
Kerosene			INR	
Diesel			INR	
Rubbish (paper, plastic etc.)			INR	
Coconut-shell			INR	
Charcoal			INR	
LPG			INR	
Other (specify)			INR	

Wood → How often are you using dry/damp wood for cooking on a scale from 1 to 5?
1= my wood is always damp
2= most of the time, my wood is damp
3= half of the time, my wood is damp and half of the time, my wood is dry
4=most of the time, my wood is dry
5=my wood is always dry

Did you cook any of your meals in the house of your neigbor who has the new stove in the past week? Yes, more than 3 times per week / Yes, 1-3 times per week / No / Not known/ No response

Thank participant for cooperation – End interview
