# Supplementary material for: Low-smoke chulha in Indian slums: study protocol for a randomised controlled trial
Source: BMC Public Health. 2017 May 16;17:454. doi: 10.1186/s12889-017-4369-6 (PMC5434517; doi:10.1186/s12889-017-4369-6)
Supplement: Supplementary file 2 — Interview guide. (PDF 152 kb) [file 12889_2017_4369_MOESM2_ESM.pdf]

Interview guide: Changes in behavior and attitude of users
For primary cook

Remarks:<br><br>1) **All answers will be recorded**<br>2) **The interviewer can ask probing questions if necessary**<br>3) The participant can always choose to give no response<br><br>The participant has signed the informed consent form and opportunity has been given to ask questions and clear up any doubts<br>*(confirm in order to continue)*

General information
Household ID (*to be filled by interviewer):*
Person ID (*to be filled by interviewer):*
Name:
Name interviewer (*to be filled by interviewer):*
Date of interview (*to be filled by interviewer):*

- What are the things you <u>don't</u> like about the stove?

- What are the things you like about the stove?

- Did you make any changes to the stove? If yes, why?

- How can the stove be improved?

- In your opinion, why do you think it is difficult for other people to adopt the new stove?

- In your opinion, why do you think other people are using the new stove?
